# A Review: Plant Carbohydrate Types—The Potential Impact on Ruminant Methane Emissions

**DOI:** 10.3389/fvets.2022.880115

**Published:** 2022-06-17

**Authors:** Xuezhao Sun, Long Cheng, Arjan Jonker, Sineka Munidasa, David Pacheco

**Affiliations:** ^1^The Innovation Centre of Ruminant Precision Nutrition and Smart and Ecological Farming, Jilin Agricultural Science and Technology University, Jilin, China; ^2^Jilin Inter-Regional Cooperation Centre for the Scientific and Technological Innovation of Ruminant Precision Nutrition and Smart and Ecological Farming, Jilin, China; ^3^Grasslands Research Centre, AgResearch Limited, Palmerston North, New Zealand; ^4^Faculty of Veterinary and Agricultural Sciences, The University of Melbourne, Melbourne, VIC, Australia

**Keywords:** fibre, greenhouse gas, livestock, feed, rumen, degradation

## Abstract

Carbohydrates are the major component of most ruminant feeds. The digestion of carbohydrates in the rumen provides energy to the ruminants but also contributes to enteric methane (CH_4_) emissions. Fresh forage is the main feed for grazing ruminants in temperate regions. Therefore, this review explored how dietary carbohydrate type and digestion affect ruminant CH_4_ emissions, with a focus on fresh forage grown in temperate regions. Carbohydrates include monosaccharides, disaccharides, oligosaccharides, and polysaccharides. Rhamnose is the only monosaccharide that results in low CH_4_ emissions. However, rhamnose is a minor component in most plants. Among polysaccharides, pectic polysaccharides lead to greater CH_4_ production due to the conversion of methyl groups to methanol and finally to CH_4_. Thus, the degree of methyl esterification of pectic polysaccharides is an important structural characteristic to better understand CH_4_ emissions. Apart from pectic polysaccharides, the chemical structure of other polysaccharides *per se* does not seem to affect CH_4_ formation. However, rumen physiological parameters and fermentation types resulting from digestion in the rumen of polysaccharides differing in the rate and extent of degradation do affect CH_4_ emissions. For example, low rumen pH resulting from the rapid degradation of readily fermentable carbohydrates decreases and inhibits the activities of methanogens and further reduces CH_4_ emissions. When a large quantity of starch is supplemented or the rate of starch degradation is low, some starch may escape from the rumen and the escaped starch will not yield CH_4_. Similar bypass from rumen digestion applies to other polysaccharides and needs to be quantified to facilitate the interpretation of animal experiments in which CH_4_ emissions are measured. Rumen bypass carbohydrates may occur in ruminants fed fresh forage, especially when the passage rate is high, which could be a result of high feed intake or high water intake. The type of carbohydrates affects the concentration of dissolved hydrogen, which consequently alters fermentation pathways and finally results in differences in CH_4_ emissions. We recommend that the degree of methyl esterification of pectic polysaccharides is needed for pectin-rich forage. The fermentation type of carbohydrates and rumen bypass carbohydrates should be determined in the assessment of mitigation potential.

## Introduction

Global warming resulting from the emissions of greenhouse gases (GHG) is a worldwide issue. Greenhouse gases emitted from anthropomorphic activities, including industrial and agricultural production, are the main driving force of global warming (1). A target for net zero emissions was set to achieve a balance between emission and removal of anthropogenic GHG by 2050 (2, 3). Methane (CH_4_) is an important gas causing greenhouse effects on the earth, and its atmospheric warming potential is 28 times that of carbon dioxide (CO_2_) (4). The global annual emissions of CH_4_ into the atmosphere are 500–600 million tonnes, and the half-life of CH_4_ in the atmosphere is 8.6 years (5). In November 2021, over 110 countries pledged to reduce global CH_4_ emissions by at least 30% in 2030 compared with 2020 (https://www.globalmethanepledge.org/). Agriculture accounts for 62% of global CH_4_ emissions from human activities, of which ruminants account for 58% of agricultural emissions (6). Enteric fermentation represents about 30–32% of total anthropogenic CH_4_ emissions in the world (7). Thus, ruminant production industries are an important source of CH_4_ emissions. Methane emissions from ruminants not only aggravate the global greenhouse effect but also cause an energy loss in livestock, accounting for 3.9–10.7% of ingested metabolic energy (8).

Although ruminants can produce CH_4_ in the hindgut, most CH_4_ is formed in the rumen (9). The feed ingested by ruminants is degraded and fermented by microorganisms in the rumen to produce products such as short-chain fatty acids (SCFA), CO_2_ and metabolic hydrogen (H_2_) (10). Short-chain fatty acids are absorbed through the rumen wall to provide energy. Metabolic H_2_ and CO_2_ are used by methanogens to produce CH_4_. In this process, CO_2_ is the carbon source, and H_2_ is the main electron donor. Four moles of hydrogen (H_2_) can produce 1 mole of CH_4_ (11). Methanogenesis is the main biochemical pathway to remove metabolic H_2_ for maintaining a very low concentration of H_2_ in the rumen (about 1 μM dissolved H_2_). If the H_2_ concentration increases, the feed degradation rate decreases. The CH_4_ emissions can be reduced by inhibiting the formation of H_2_ from fermentation or promoting alternative pathways of H_2_. A few species of methanogens use alcohols as electron donors to participate in the formation of CH_4_, but most methanogens use H_2_ and formate as electron donors (11). Although there are other methanogenic microorganisms in the rumen, methanogens are absolutely dominant. Methanogens belong to Archaea (12). There are 10^7^-10^9^ cells of methanogenic archaea per mL of rumen fluid (13). There are three main types of Archaea in the rumen. Cluster A is the most important, mainly including *Methanobrevibacters*; Cluster B mainly *Methanosphaera* and Cluster C the remainder. Clusters A and B account for 84% of the total number of Archaea in the rumen (12). The pH range suitable for the growth of methanogens is 6.0–7.5, with the bottom limit of 5.5–6.5. Other microorganisms in the rumen, such as protozoa, have an indirect impact on CH_4_ emissions. Protozoa use starch, cellulose, hemicellulose, pectin and soluble sugar to produce SCFA and metabolic H_2_. The metabolic H_2_ is converted to CH_4_ by methanogens attached to the surface of protozoa (14).

In temperate countries with animal agriculture based on pastoral systems, pastures are dominated by perennial ryegrass, but mixtures of ryegrass with clover or herbs and grazing forage crop monocultures have become more common to improve animal performance, fill pasture feed gaps, and reduce environmental impact (15, 16). These alternative pasture species contain a wide range of non-structural carbohydrates (NFC) and generally have a greater ratio of NFC to structural carbohydrates than ryegrass pasture. We postulate that the reported differences in CH_4_ emissions between ryegrass and alternative forages (15, 17, 18) might be due to their difference in carbohydrate composition and rate of digestion, as it is known that carbohydrates affect CH_4_ emissions (19).

The hydrolysis of di-, oligo- and poly-saccharides in the rumen into monosaccharides is a complex process with a large number of enzymes involved. However, it is interesting to note that no H_2_ is produced, and thus, no CH_4_ is formed during this process (11). On the other hand, the fermentation of monosaccharides to pyruvate and then into SCFA such as acetate and butyrate results in H_2_ formation, which is mainly converted into CH_4_ by ruminal methanogens. The type of SCFA produced from the fermentation of pyruvate depends on the ruminal microbial community structure and ruminal environment (11, 20, 21), which can affect CH_4_ emissions (21, 22). The dissolved H_2_ concentration in the rumen is a key factor determining the SCFA formation pathways and end-products (20). Feeding different dietary carbohydrate fractions often results in different ruminal fermentation and passage rates (23), ruminal pH, rumen buffering capacity, and different (acetate + butyrate)/propionate ratios (21, 24). Therefore, changing forage carbohydrate composition might be an effective way to mitigate CH_4_ emissions. However, to the best of our knowledge, few systematic reviews are performed in this area although some reviews have more or less touched upon carbohydrates [e.g., (10, 25–28)], despite many study results being published over the last few decades. Therefore, the aim of this review was to summarise published data on carbohydrate types and their molecular structure present in ruminant feeds, with an emphasis on fresh forages and their role in enteric CH_4_ emissions. The literature for this review was searched using SCOPS with methane, ruminant (or rumen, methanogen) and carbohydrate (including terms in this category such as monosaccharide, disaccharide, polysaccharide, pectin, starch, fructan, etc.) and with methane and temperate forage (including the common or scientific names of temperate forages) as keywords and the date range set between 2000 and 2022.

## Fresh Forage Carbohydrate Composition and CH_4_ Emissions From Ruminants

**Figure 1:**
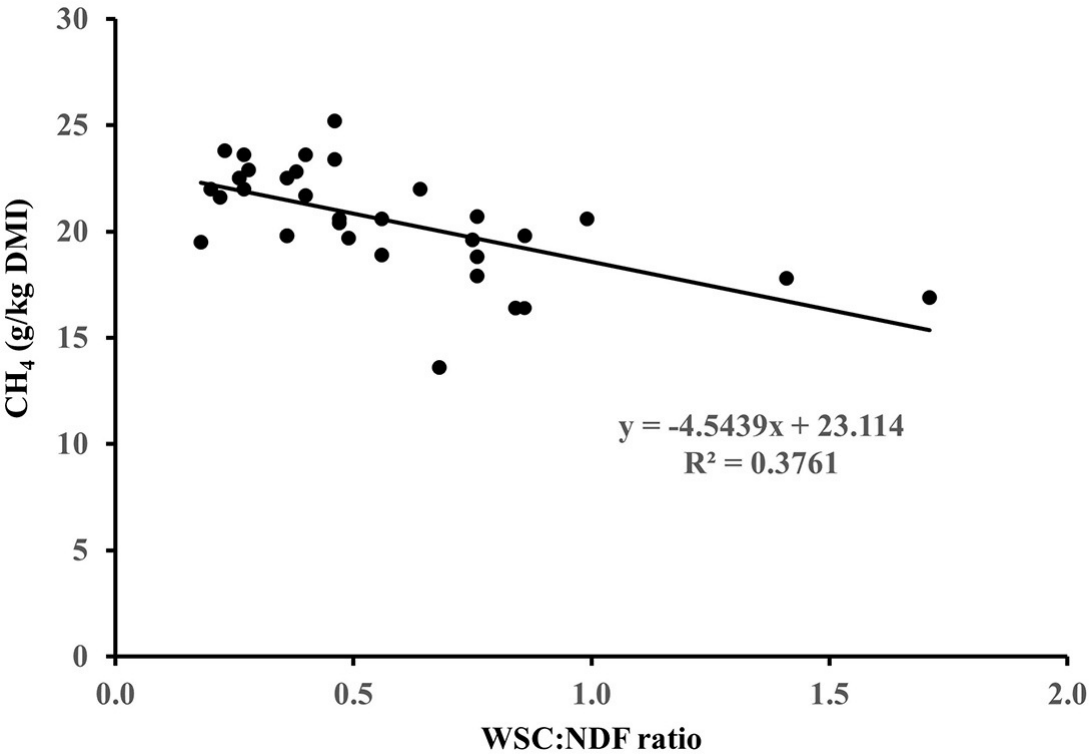
Relationship between CH_4_ yield (g/kg dry matter intake; DMI) and forage water soluble carbohydrate (WSC): neutral detergent fibre (NDF) ratio in sheep fed different fresh forages.

Improved pastures in pastoral animal production systems are generally dominated by perennial ryegrass (*Lolium perenne* L.) (29). Perennial ryegrass pasture composition was previously found to have only minor effects on CH_4_ emissions from sheep and cattle (29–31). However, mixing pastures with clover or herbs or grazing forage crop monocultures is increasing. These alternative forage species have a much wider range of carbohydrate types and compositions than perennial ryegrass. A series of trials with sheep fed fresh alternative forages *vs*. perennial ryegrass have been performed (15, 32), and the detailed carbohydrate composition of the forage eaten was analysed by conventional gravimetric and spectrophotometric methods (Table 1). Carbohydrate fractions analysed included water soluble carbohydrates (WSC) after hot-water extraction (consists of mono-, di-, and oligo- saccharides), pectin using meta-hydroxydiphenyl (44), and fibre residue after neutral detergent (NDF, consists of hemicellulose, cellulose and lignin) and acid detergent (ADF, consists of cellulose and lignin) extraction (45). Hemicellulose concentration was estimated as NDF – ADF, cellulose concentration as ADF – the residue left after sulphuric acid extraction of ADF, and total NFC as 1 – ash – crude protein – lipids – NDF. Starch concentration was assumed to be negligible in these fresh forages. The studies showed that only sheep fed forage brassicas had consistently lower CH_4_ emissions than sheep fed perennial ryegrass pasture (15, 35, 37, 46), while sheep fed forage chicory (33, 34) or white clover (17) had similar CH_4_ emission to sheep fed perennial ryegrass pasture (Table 1). Although there was a moderate negative relationship (*R*^2^ = 0.38) between CH_4_ yield [g/kg dry matter intake (DMI)] and WSC: NDF ratio (Figure 1), the forage carbohydrate composition did not fully explain differences in CH_4_ emissions (32, 47). Therefore, other potential mechanisms behind lower CH_4_ emissions in sheep fed forage brassicas were investigated, including chemical and digestive parameters like hydrogen sinks (nitrate and sulphur), plant secondary compounds (S-methyl cysteine sulfoxide and glucosinolates), rumen fermentation profiles (*i.e.*, SCFA profile), solid and liquid rumen turnover rates, total tract digestibility and free triiodothyronine in blood (related to digesta fractional passage rate) (35, 37, 46), but none of these individual variables had a strong relationship with CH_4_ emissions (47). Therefore, the full mechanisms for the CH_4_ mitigation properties of forage brassicas remain unclear to date. Sun (48) proposed a new hypothesis based on a literature review to explain the low enteric CH_4_ emission observed from ruminants fed forage brassica. According to the hypothesis, glucosinolates, a secondary metabolite widely present in forage brassica or their breakdown products, can stimulate the secretion of free triiodothyronine in ruminants. This may change rumen physiology by reducing the mean retention time of digesta in the rumen, ultimately reducing enteric CH_4_ emissions.

**Table 1:** Summary of methane measurements (measured using respiration chambers) in sheep fed different fresh temperate forages of varying chemical composition and crude carbohydrate fractions.

	**DM**	**Ash**	**CP**	**Lipid**	**WSC**	**Pectin**	**NFC**	**NDF**	**ADF**	**ADL**	**Hemi-cellulose**	**Cellulose**	**WSC:NDF**	**NFC:NDF**	**RFC:SC**	**DMI**	**CH_**4**_**	**References**
	**g/kg DM**												**Ratio**			**g/d**	**g/kg DMI**	
White clover Exp1	166	95	255	24	98	65	350	276	183	86	93	97	0.36	1.27	0.86	930	19.8	(17)
Ryegrass Exp1	172	136	192	31	114	7	197	444	230	37	214	193	0.26	0.44	0.30	1,100	22.5	
White clover Exp2	162	89	214	22	125	65	406	269	183	86	86	97	0.46	1.51	1.04	1,160	23.4	
Ryegrass Exp2	163	97	125	28	164	7	335	415	220	18	195	202	0.40	0.81	0.43	1,190	21.7	
Chicory	89	144	117		107	65		281	213	80	68	133	0.38	1.63	0.86	772	22.8	(33)
Ryegrass	154	84	85		115	6		499	275	22	224	253	0.23	0.67	0.25	752	23.8	
Kale	141	139	167	34	173	80	459	201	129	57	72	72	0.86	2.28	1.76	866	19.8	(34)
Rape	126	140	193	34	196	89	399	234	163	63	71	100	0.84	1.71	1.67	899	16.4	
Swedes	94	92	162	11	301	69	559	176	121	51	55	70	1.71	3.18	2.96	792	16.9	
Turnips	101	149	130	17	238	94	464	240	180	63	60	117	0.99	1.93	1.88	874	20.6	
Ryegrass	176	154	150	36	106	10	124	536	277	30	259	247	0.20	0.23	0.23	946	22.0	
Chicory	119	196	114		153	75.2		239	188		51		0.64	1.89	4.47	890	22.0	(35)
Ryegrass	165	127	197		114	10.4		423	218		205		0.27	0.60	0.61	869	23.6	
White clover P1	163	99	262	26	102	68	327	286	190	89	96	101	0.36	1.14	0.86	940	19.8	(17)
Ryegrass P1	173	140	196	32	117	8	177	455	235	38	220	197	0.26	0.39	0.30	1,120	22.5	
White clover P2	162	92	222	23	129	68	383	280	190	90	90	100	0.46	1.37	1.03	815	25.2	
Ryegrass P2	163	99	128	29	168	7	319	425	226	19	199	207	0.40	0.75	0.43	835	23.6	
White clover P3	160	97	220	23	106	63	364	296	203	69	93	134	0.36	1.23	0.74	910	22.5	
Ryegrass P3	184	102	117	27	126	7	288	466	237	18	229	219	0.27	0.62	0.30	930	22.0	
White clover	160	97	220	23	169*		364	296	203		93			1.23		1,140	22.5	(17)
Ryegrass	184	102	117	27	133*		288	466	237		229			0.62		1,120	22.0	
Ryegrass	189	85	110		252			512	250		262		0.49			1,080	19.7	(36)
Rape P1	131	148	215	34	142	76	394	209	161	38	48	123	0.68	1.89	1.27	862	13.6	(37)
Ryegrass P1	148	158	181	41	83	9	156	464	242	27	222	215	0.18	0.34	0.21	792	19.5	
Rape P2	142	83	158	34	240	75	555	170	123	37	47	86	1.41	3.26	2.37	896	17.8	
Ryegrass P2	198	99	160	35	123	11	261	445	231	17	214	214	0.28	0.59	0.31	929	22.9	
Ryegrass	145	107	202		112			507	257		250		0.22			1,130	21.6	(38)
Ryegrass HSG	167	96	134	39	249		288	443	261	16	182	245	0.56	0.65	0.58	850	18.9	(39)
Ryegrass CRG	166	91	142	41	311		318	408	251	16	157	235	0.76	0.78	0.79	825	20.7	
Ryegrass TRG	130	114	156	44	202		259	427	261	18	166	243	0.47	0.61	0.49	825	20.6	
Ryegrass	203	67	114					526	254		272					950	19.1	(40)
CRG P1	167	96	134	39	249		288	443	261	16	182	245	0.56	0.65	0.58	820	20.6	(29)
HSG P1	166	91	142	41	311		318	408	251	16	157	235	0.76	0.78	0.79	870	18.8	
TRG P1	130	114	156	44	202		259	427	261	18	166	243	0.47	0.61	0.49	740	20.4	
CRG P3	209	90	141	42	308		316	411	217	13	194	204	0.75	0.77	0.77	920	19.6	
HSG P3	207	87	141	42	302		330	400	204	14	196	190	0.76	0.83	0.78	900	17.9	
TRG 3	193	95	126	39	330		358	382	206	10	176	196	0.86	0.94	0.89	900	16.4	
Ryegrass	203	109	124					505	248	21	257	227				1,390	18.5	(41)
Chicory	103	175	142					353	208	63	145	145				1,740	14.3	
Vegetative ryegrass Exp1	205	79	102				265	531	285	31	246	254		0.50		874	23.1	(42)
Mature ryegrass Exp1	284	57	64				265	610	320	37	290	283		0.41		891	21.4	
Vegetative ryegrass Exp2	254	96	118				195	565	280	47	285	233		0.35		922	20.4	
Mature ryegrass Exp2	375	55	54				209	674	373	50	301	323		0.31		873	17.2	
Vegetative ryegrass Exp3	143	125	195				213	431	241	18	190	223		0.49		747	18.1	
Lowland ryegrass	162	73	123		219			528	268		260					910	19.1	(43)
Hill ryegrass	211	42	96		135			657	316		341					630	19.3	

*ADF, acid detergent fibre; ADL, acid detergent lignin; CP, crude protein; CRG, conventional diploid ryegrass; DM, dry matter; DMI, dry matter intake; Exp1, Exp2 and Exp3, experiments 1, 2 and 3; HSG, high sugar diploid ryegrass; NDF, neutral detergent fibre; NFC, non-fibre carbohydrates (calculated as 1000 – ash – CP – lipid - NDF); P1, P2 and P3, experimental periods 1, 2 and 3; RFC, readily fermentable carbohydrates [water soluble carbohydrates (WSC) + pectin]; SC, structural carbohydrates [hemicellulose (NDF–ADF) + cellulose (ADF–ADL)]; TRG, tetraploid ryegrass; WSC, water soluble carbohydrates*.

^*^The sum of WSC and pectin.

The forage effects of NFC on ruminant performance and CH_4_ emissions have been studied extensively over the last two decades. For example, high levels of NFC in the diet can be delivered by feeding high sugar grasses (HSG). In a study by Kim et al. (49), lambs fed fresh HSG emitted 17% less CH_4_ than lambs fed conventional perennial ryegrass (19.3 vs. 23.3 g/kg DMI) and when these ryegrasses were mixed with white clover, such CH_4_ emission differences remained (18.6 vs. 21.2 g/kg DMI). In New Zealand, sheep fed an HSG cultivar or a tetraploid ryegrass cultivar were found to have a lower CH_4_ yield than sheep fed a conventional diploid ryegrass cultivar (Table 1) (29, 39). However, Staerfl et al. (50) found similar CH_4_ yield (19.4 and 20.3 g CH_4_/kg DMI, respectively) in lactating Holstein-Friesian dairy cows fed HSG or conventional ryegrass hay [492 vs. 491 g NDF, 224 vs. 101 g NDF, 193 vs. 103 g WSC, and 129 vs. 82 g ethanol soluble carbohydrates per kg of dry matter (DM), respectively]. Therefore, the effect of increasing dietary NFC concentration in ryegrass or by feeding alternative forages, on CH_4_ emissions has been variable.

## Carbohydrate Compositions

**Figure 2:**
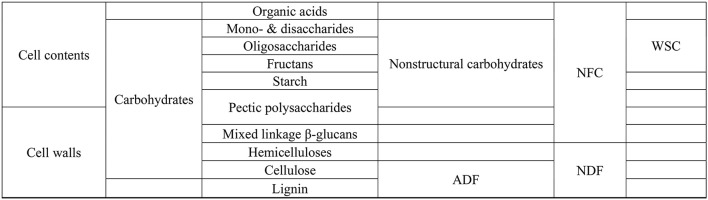
Plant carbohydrates. ADF, acid detergent fibre; NDF, neutral detergent fibre; NFC, non-fibre carbohydrates; WSC, water soluble carbohydrates. Adapted from Hall et al. (51).

The chemical characterisation of carbohydrates in feeds/forages in the previous sections was performed using conventional gravimetric and spectrophotometric methods, which do not provide a detailed characterisation of the types (Figure 2) and molecular structure of plant carbohydrates. *In vitro* studies have provided evidence that different types of carbohydrates change CH_4_ emissions (52–55). Carbohydrates in plants are present as mono-, di-, oligo- and- poly-saccharides (56) and can be linked with other compounds in the plant such as protein and lignin to form the plant cell wall structures.

### Free Monosaccharides

Glucose and fructose are present as free monosaccharides in plants (57, 58) at concentrations generally below 10 g/kg DM, although they can be up to 61 g/kg DM in spring grass (57). Glucose can account for 20–90% of total non-structural carbohydrates in the leaves of tropical grasses (59) and 11–36 g/kg DM (60) and 10–20 g/kg DM (58) in some cool-season grasses. Fructose is not detectable or is only present in very small amounts in the leaves of tropical grasses (59) and between 8 and 22 g/kg DM (60) and 5–12 g/kg DM (58) in the leaves of cool-season grasses. Free sugars are estimated to degrade in the rumen at a rate of 40–60%/h (61).

### Monosaccharides in Di-, Oligo-, and Poly-Saccharides

Monosaccharides are the basic units of disaccharides, oligosaccharides and polysaccharides, and in plants include pentoses (5-carbon sugars) such as xylose and arabinose, and hexoses such as glucose, galactose, rhamnose, mannose, fructose, glucuronic acid, and galacturonic acid. Many of these can occur in a variety of different polysaccharides. For example, in lignified secondary cell walls of dicotyledons, xylose occurs mostly in xylans, but in primary walls of dicotyledons, it occurs mostly in xyloglucans, with small amounts in pectic polysaccharides. Similarly, in primary cell walls of dicotyledons arabinose, galactose, rhamnose, and galacturonic acid occur mostly in pectic polysaccharides. These associations result in different rates and extent of digestion of monosaccharides from the polysaccharides. The ratio of xylose to arabinose has been suggested as an indicator of the potential digestion of forage cell walls (62). This would apply particularly to dicotyledonous forages, but even in grasses where heteroxylans containing arabinose occur in both primary and lignified secondary cell walls, the proportion of arabinose in these polysaccharides is greater in primary cell walls, which also contain pectic polysaccharides containing arabinose (44). Therefore, it is important to know the monosaccharide composition of carbohydrates in forages.

Di- and- oligo-saccharides, non-fibre polysaccharides and soluble fibre are part of NFC, which are rapidly degraded in the rumen (20–60%/h) and to a large extent (61) but vary in concentrations, monosaccharide composition and structure (see next section), which might affect CH_4_ emissions differently.

### Di- and- Oligo-Saccharides

The main disaccharide present in forages and feeds is sucrose, and the main oligosaccharides are raffinose, stachyose and verbascose, but the contents of sucrose (<40 g/kg DM) and oligosaccharides (<40 g/kg DM) are generally low across a range of feeds including whole plant cereals (57). However, the concentration of sucrose in grasses reached up to 62 g/kg DM in the leaves of *Festuca arundinacea*, between 24 and 42 g/kg DM in *Agropyron* (red), *L. perenne, Dactvlis glomerata, Phleum pratense*, and *Bromus inermis*, but was <9 g/kg DM in *Bromus tectorum* (60), 8–18 g/kg DM in *F. arundinacea, Lolium multiflorum* × *F. arundinaceae, Thinopyrum ponticum, Thinopyrum intermedium*, and *B. inermis* (58) and 24–42 g/kg DM in *P. pratense* (63). The simple sugars in fresh fodder beet underground storage organs and leaves consist of 15–20% sucrose (64).

### Fructans

Fructans are present in the vegetative parts of temperate grasses ranging from 19 to 383 g/kg DM, typically 25–50 g/kg DM (58). Fructans have a linear chain of β-2,6-fructosyl-fructose residues and can have a branched structure by both β2-1 and β2-6 linkages in some plant species. The side and branch chains of fructans have a α-1,2-linked residue as a terminal unit in most cases, which is a non-reducing end (65, 66). Fructans with a low degree of polymerisation can be extracted with boiling 80% ethanol, and fructans with a high degree of polymerisation can be extracted with boiling water (67). Fructans are rapidly and completely fermented in the rumen (68). In the 1970s, a high fructan content was found in perennial ryegrass plants of specific populations, leading to the breeding of cultivars called generically “high sugar grass” (69), which have received considerable attention for their putative benefits to animal production (29, 69, 70). Inulin is also a fructan, sometimes called inulin-type fructan, but it is a linear β 2-1 fructan with a basic unit of α-D-glucose linked to 1-2 β-D-fructose β (1, 5) bond (66). Inulin is widely present in other forage crops like the tubers of Jerusalem artichoke (*Helianthus tuberosus*) (71) and the roots of chicory (66).

### Starch

Starch is a carbohydrate composed of glycosidically linked glucose units and composed of two types of polysaccharides: linear amylose and branched amylopectin. Amylose has a linear chain only, and the length of the chain is several hundred glucose residues (72). Amylose occasionally contains α-1,6 linked glucose. Amylopectin has a chain of α1,4-linked glucose residues with side chains linked by α1,4,6-linked glucose residues (72). The side chain sometimes has terminal glucose which is a reducing sugar (72, 73). A large number of the macromolecular chains in starch granules are organised in crystalline structures, with three patterns of crystalline structures occurring: A-type pattern common in cereal starches, B-type pattern present in some tuber starches and cereal starches, and C-type pattern is common in legume starches. B-type crystalline starches with larger granules are more resistant to enzymatic breakdown than A-type crystalline starch with small granules (74). Starch is rapidly degraded in the rumen (20–40%/h; 51), with the physical structure surrounding starch granules being the main barrier to degradation (73). Starch concentration in vegetative parts of forages is very low, typically ranging from 10 to 33 g/kg DM (58), with 40 g/kg DM in the leaves of *Lolium temulentum* (75), 34 g/kg DM in lucerne leaves, 3 g/kg DM in lucerne stems, 4 g/kg DM in timothy hay (76), 2–3 g/kg DM in timothy leaves (63) and 5–17 g/kg DM in forage brassicas (Sun et al., unpublished data). Starch is the main storage polysaccharide in grains and some tubers such as potatoes and sweet potatoes, but not in the underground storage organs of swedes, turnips, and fodder beet.

### Pectic Polysaccharides

Pectic polysaccharides are present in the primary cell walls of all forage plants and are located particularly in the middle lamella (77). The pectic polysaccharides in the primary walls of grasses (family Poaceae) have a similar structure to those in dicotyledonous primary walls (78), but they occur in much lower proportions in Poaceae (Table 1). The principal monosaccharide components of pectins are galacturonic acid, rhamnose, arabinose and galactose (79). There are three main types of pectic polysaccharide domains: homogalacturonan (HG), rhamnogalacturonan I (RG I), and rhamnogalacturonan II (RG II) (80) and a small amount of a fourth domain, xylogalacturonan (XGA), occurs in some walls (44, 78). The XGA is composed of a backbone of 4-linked α-D-galactosyluronic acid residues to which xylose residues are attached at the C(O)3 position of about half of the galacturonic acid residues (78).

The HG is a linear homopolymer of 4-linked α-D-galactosyluronic acid residues. Methyl-esterification of galactosyluronic acid residues often occurs at the C-6 carboxyl group; acetyl groups are also present at C-2 and/or C-3 in some plants (79). Two HG chains with blocks of more than 10 unesterified galacturonic acid (GalA) residues can form a dimer via Ca^2+^-cross-linking (80). RG I consists of a backbone of alternating (1, 7)-linked α-D-galacturonic acid and (1, 5)-linked α-L-rhamnose residues and has several different side-chains attached to the C4 of rhamnose. These side-chains are mainly composed of arabinose and galactose chains (arabinans and galactans, respectively) linked by (1, 8)-linkages and by (1, 7)-linkages, respectively (81). Type I arabinogalactan side-chains have (1, 7)-β-galactan chains with limited substitution by short arabinose-containing oligomers (82). RG II is quantitatively a minor constituent of primary cell walls in dicotyledons (83), and it has been isolated from the primary cell walls of the Poaceae. It is released as low-molecular-weight polysaccharides, containing about 30 different glycosidic linkages with at least 12 different glycosyl residues, after treating with endo-polygalacturonase (78).

### Cellulose

Cellulose, a simple linear polymer of (1, 7)-linked β-D-glucosyl residues, is the most abundant plant polysaccharide and forms the basic structure of all plant cell walls (84). Cellulose occurs as crystalline microfibrils about 3 nm in diameter containing 18 cellulose molecules aligned side to side (85, 86). In secondary lignified cell walls, some of the cellulose microfibrils may aggregate into structures known as macrofibrils (87). Primary cell walls contain ~20–30% cellulose, and lignified secondary walls contain ~40–60% cellulose (88). Cellulose typically accounts for about 35~50% of plant dry matter (89).

### Other Non-Cellulosic Polysaccharides (“Hemicelluloses”)

Other non-cellulosic polysaccharides have traditionally been called hemicelluloses and comprise a group of polysaccharides that are extracted from cell walls with alkaline solution after initial treatments with water and chelating reagents (90). They include xyloglucans, heteroxylans, heteromannans, and β-glucans and are bound to cellulose by hydrogen bonds. Their structures vary greatly in the walls of different cell types and different plant species. Xyloglucans (XGs) are the principal other non-cellulosic polysaccharides of dicotyledonous primary cell walls (91), accounting for up to 20–25% of the primary cell walls (92). The main backbone is composed of 4-linked β-D-glucosyl residues, most of which are linked to α-D-xylosyl residues of side chains with α-(1, 9)-glycosidic linkage and these xylosyl residues can be linked to fucose, galactose, and less commonly arabinose (92).

Fucogalactoxyloglucans have been found in the primary walls of most dicotyledonous plants except for those of solanaceous plants (e.g., potato), which contain arabinoxyloglucans (93). In the walls of the monocotyledon family Poaceae (grasses), the xyloglucans do not contain arabinose and fucose (94). The content of xyloglucans in members of the Poaceae family is small, accounting for only 2–5% of the walls (95).

Heteroxylans include arabinoxylans, glucuronoxylans, and glucurono-arabinoxylans. They have a backbone of 4-linked xylose residues with short side chains of arabinose, glucuronic acid and 4-O-methyl-glucuronic acid residues (96). The xylose residues in the backbone may be O-acetylated, and in the Poaceae the arabinose residues may be esterified with ferulic or *p*-coumaric acid (96, 97). Heteroxylans are present in small proportions in the primary walls of dicotyledons, but they are abundant in the Poaceae. Cellulose microfibrils in the walls of Poaceae may be interlocked mainly by glucuronoarabinoxylans (GAXs). Unbranched GAXs are cross-linked with each other or to cellulose via hydrogen bonds. Branched GAXs are unable to form crosslinking between two GAXs or GAX to cellulose since arabinose and glucuronic acid side groups prevent the formation of hydrogen bonds (95). Galactoarabinoxylans have 4-linked xylan backbone with α-L-arabinofuranose residues and/or short chains containing arabinose and galactose attached at C3- or C2- positions. They are found in perennial ryegrass (98, 99) and cocksfoot grass (*Dactylis glomerata*) leaves (98).

Heteromannans galactoglucomannans (GGM) are found widely in a small amount, and they have a backbone of (1, 7)-linked β-D-mannose and β-D-glucose residues.

(1,3:1,4)-β-Glucans or written as(1 → 3), (1 → 4)-β-glucans are also known as mixed-linked glucans or β-glucans and consist of unbranched and unsubstituted chains of (1,3)- and (1,4)-β-glucosyl residues with varying ratio of (1,4)-β-D-glucosyl residues to 1,3)-β-D-glucosyl residues (100). They have been found in the primary cell walls of Poaceae, and contain about 30% 3-linked residues and 70% 4-linked residues (101). In cereal grains, they occur particularly in the walls of the aleurone, and starchy endosperm and their contents vary markedly, largely depending on species (102), and these polysaccharides are particularly abundant in barley (103). (1,3:1,4)-β-Glucans are thought to form a gel-like matrix in cell walls between the reinforcing cellulose microfibrils (102).

## Effects of Carbohydrate Types on Methane Emissions From Ruminants

### Monosaccharides

Czerkawski and Breckenridge (52) conducted *in vitro* rumen simulation technique (RUSITEC) incubations with 26 carbohydrates. These carbohydrates resulted in similar CH_4_ production per unit of carbohydrate fermented, except for rhamnose. Rhamnose resulted in distinguishable CH_4_ production, but this monosaccharide is only a minor component in plants (44). However, carbohydrates differed in their extent and rate of fermentation: glucose, fructose and sucrose were fermented rapidly; L-arabinose, xylose galactose, and mannose were fermented at an intermediate rate; and glucuronic acid, galacturonic acid, and fucose fermented at a slow rate (52). Carbohydrates with a slow fermentation rate have a higher probability of escaping the rumen without being fermented (104), which could result in lower CH_4_ emissions. Pacheco et al. (105) speculated that the escape of soluble cell contents could explain why CH_4_ produced per unit of digestible organic matter was less in forages with a greater content of water.

### Disaccharides

Sucrose concentration is generally low in forages, but it can be high in some feeds, for example, as the main storage carbohydrate in some roots like those of fodder beet. Its fractional fermentation rate in the rumen was about 1,200–1,404%/h (106), which is much higher than for starch [e.g., 30%/h for barley starch (107)]. In a sheep trial conducted by Huhtanen and Robertson (108), with 400 g/kg DM of sugar-beet pulp replaced by sucrose, maize starch or xylose, CH_4_ emissions were similar for sheep supplemented with sucrose, maize starch or xylose [6.6% of gross energy (GE)], but lower than those fed sugar-beet pulp (7.1% of GE intake). Sucrose was more rapidly fermented than steam-flaked maize starch *in vitro* at two pre-determined pH levels (pH 6 and 7) (109). Methane emissions per unit of degraded organic matter (OM) were similar for sucrose and starch at pH 6, but more CH_4_ was produced from sucrose than from starch at a pH of 7. At pH 6, sucrose led to a higher molar proportion of propionate and less butyrate than starch, while at pH 7, the fermentation profile was similar for both sucrose and starch. At both levels of pH, sucrose produced more CH_4_ and more SCFA (109). These findings suggest that pH in the rumen will interact with carbohydrate type leading to varying levels of CH_4_ production in response to carbohydrate supplementation.

Golder et al. (110) found that supplementation of the diet with crushed triticale grain decreased rumen pH and increased total SCFA, acetate, butyrate and propionate concentrations compared with the control and these changes were even more extreme when triticale grain plus fructose were supplemented. Although CH_4_ emissions were not measured, the large drop in rumen pH suggests a reduction in CH_4_ emissions could be expected.

### Pectic Polysaccharides

Pectic polysaccharides present in forage are methyl esterified to varying degrees. The hydrolysis of methyl esters from pectic polysaccharides produces methanol (CH_3_OH), which is stoichiometrically converted to CH_4_ as 4CH_3_OH → 3CH_4_ + CO_2_ + 2H_2_O. The theoretical molar ratio of CH_4_ produced from methanol (CH_4_/CH_3_OH) is 0.75. When pectin or methanol was infused into the rumen of sheep, the measured conversion ratio was 0.77, suggesting all methanol infused was converted to CH_4_ (111). Thus, forages containing large amounts of highly methyl-esterified pectic polysaccharides might result in higher CH_4_ emissions.

Poulsen et al. (54) reported no differences in CH_4_ production among sugar beet pectin, maize starch, wheat starch and chicory inulin incubated *in vitro* (batch culture), but the extent of fermentation may change the final CH_4_ production. Nevertheless, pectin resulted in a higher molar proportion of acetate, and a lower proportion of butyrate and inulin lowered acetate and increased butyrate, compared with starch from either maize or wheat. These differences in SCFA suggested that different amounts of hydrogen were produced and consequently would have led to different amounts of CH_4_ released per unit of fermented substrates. In the RUSITEC-based study by Zhao et al. (112), CH_4_ emissions were lower with inulin than with starch, and inulin resulted in a reduced molar proportion of acetate and acetate: propionate ratio and increased butyrate proportion, which suggests a potential for reduced CH_4_ emissions by feeding inulin. Consisted with this result, rumen fermentation of fructose, which is a product of inulin degradation in the rumen, resulted in less acetate and more butyrate compared with starch in steers (113).

In pectic polysaccharides, HG is a major component and has a backbone of galacturonic acid residues esterified with methyl groups to different degrees. The cleavage of methyl groups from HG may release methanol which is used for CH_4_ formation by some microbes (111). Geerkens et al. (114) compared mango peels rich in pectin, de-pectinised mango peels, apple pectin and citrus pectin *in vitro* (batch culture) mixed with hay (Table 2). Mango peels produced more CH_4_ than hay when expressed as either CH_4_/gas production (GP) or CH_4_/SCFA, while de-pectinisation of mango peels resulted in reduced CH_4_, suggesting that pectin may cause an increase in CH_4_ production. However, CH_4_/GP dropped, and CH_4_/SCFA was similar or dropped when citrus or apple pectin was incubated compared with hay. This suggests that the structure of pectic polysaccharides affects CH_4_ formation. This result is different from the finding of Czerkawski and Breckenridge (52) in which the degree of methyl esterification was not considered. When high and low degrees of methyl esterification were compared, low esterified pectin produced less CH_4_ than high esterified pectin for both apple and citrus pectins (114). This supports the hypothesis that methyl group cleavage from pectin leads to increased CH_4_ formation, with methanol as an intermediary.

**Table 2:** Total gas, methane (CH_4_) and short-chain fatty acid (SCFA) productions of pectic substrates after 24 h *in vitro* incubation [adapted from Geerkens et al. (114)].

	**Substrate**						
	**Hay**	**MP**	**dep MP**	**AP HE**	**AP LE**	**CP HE**	**CP LE**
Gas production (GP; mL/100 mg DM)	23.6	36.5	29.2	41.0	40.1	43.7	40.1
CH_4_ production (mL/100 mg DM)	5.48	9.01	6.31	7.72	6.61	8.78	7.53
CH_4_/GP (mL/mL)	0.232	0.247	0.216	0.188	0.165	0.201	0.188
Total SCFA production (μmol/100 mg DM)	475	684	533	700	702	768	745
Acetate/total SCFA (mmol/mmol)	68.1	68.6	70.6	81.9	83.6	84.1	83.8
Propionate/SCFA (mmol/mmol)	24.0	22.9	23.4	14.8	13.4	12.8	13.0
Butyrate/SCFA (mmol/mmol)	7.0	8.0	5.4	2.9	2.6	2.5	2.6
CH_4_/SCFA (mL/mmol)	11.5	13.2	11.8	11.0	9.4	11.4	10.1

*DM, dry matter; MP, mango peels; dep MP, depectinised MP; AP, apple pectin; CP, citrus pectin; HE, high-esterified; LE, low-esterified*.

### Starch

Between 22 and 94% of dietary starch eaten is digested in the rumen depending on total starch intake and starch source (115). The size and structure of starch granules and the pattern of enzyme-resistant crystalline starches may affect their degradation rate in the rumen (73). For example, starch from potatoes is degraded faster than that from barley and oats (115).

Dairy cows fed diets containing lucerne hay and concentrate (45:55 w/w) based on wheat grain had lower CH_4_ yield (11.1 g CH_4_/kg DMI) compared with cows fed concentrates based on maize grain (19.5 g/kg DMI) (116). Average daily ruminal pH was similar between cows fed either type of grain, but the duration when ruminal pH was below 6 was twice as long for wheat grain supplemented cows compared with maize grain supplemented cows. Wheat and maize have different starch structures, leading to faster degradation for wheat starch than for maize starch with values of 103.8%/h for wheat grain and 8.1%/h for maize grain reported by Moharrery et al. (115). The faster degradation rate of wheat starch might result in a longer duration of pH below 6, which in itself may inhibit methanogen activity and therefore contribute to lower CH_4_ emissions in cows fed wheat grain as found by Moate et al. (116). Lactating dairy cows fed diets based on grass-clover/maize silage (60% diet DM) and either maize cob silage, highly rumen digestible rolled barley grain or sodium hydroxide wheat grain, with a high rumen escape starch (at 25% of diet DM), had similar CH_4_ yield and total tract starch digestibility (117).

Besides starch type, grain processing also affects the degradation rate of starch in the rumen (73), which might alter the rumen fermentation profile and rumen pH and could therefore change CH_4_ emissions. Lactating dairy cows fed diets with 60% grass silage and 40% concentrate containing either slowly degradable native maize grain (5%/h) or rapidly degradable gelatinised maize grain (16%/h) at either 270 or 530 g/kg concentrate had similar CH_4_ yield and rumen pH but an increased rate of starch fermentation and increased level of starch reduced CH_4_ produced per unit of estimated rumen-fermentable organic matter (118).

**Figure 3:**
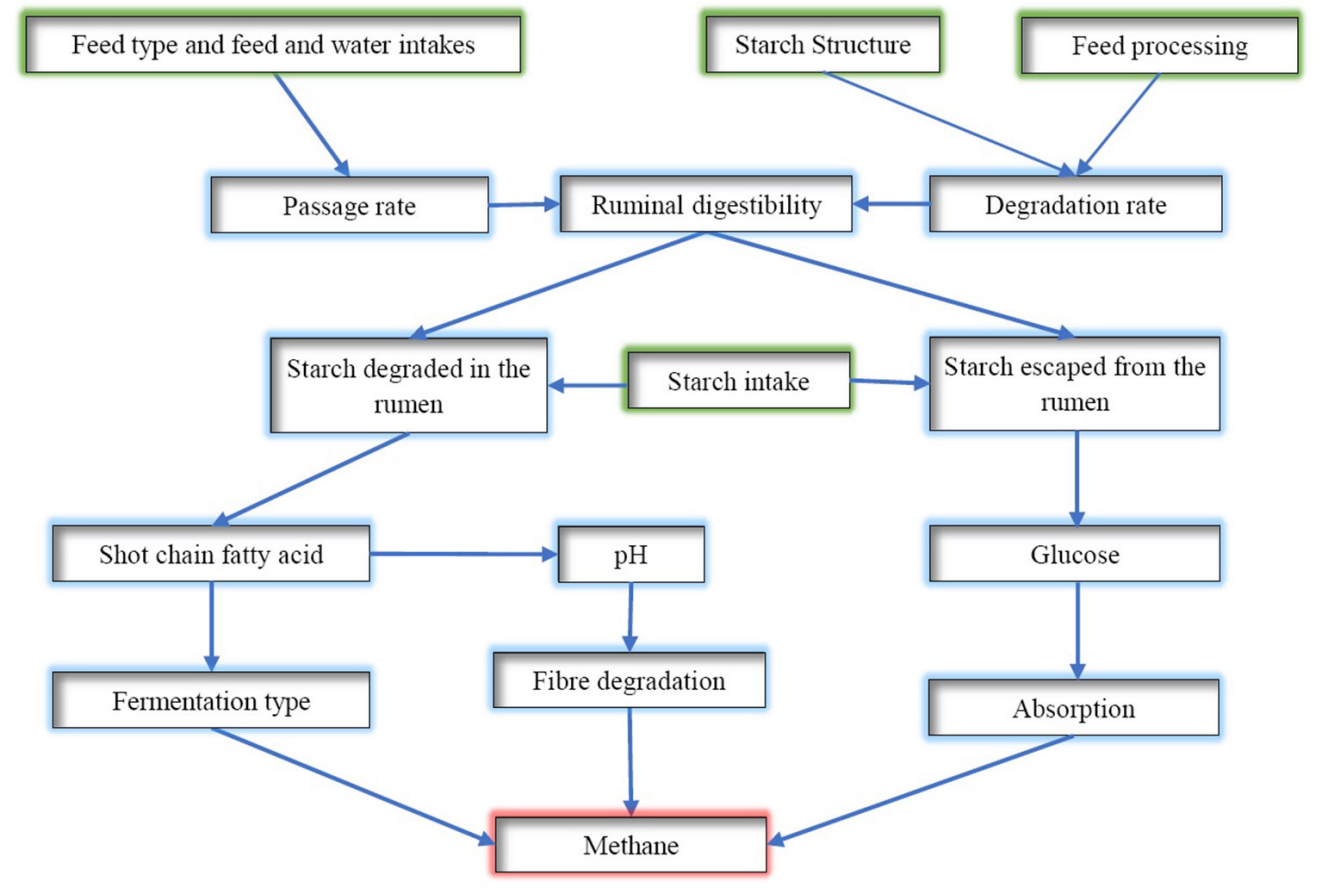
A diagram of starch digestion in the rumen and its effects on methane emissions.

Thus, the amount of starch digested in the rumen and the rate of digestion will affect the rumen fermentation pattern and rumen pH, whilst starch escaped from the rumen has little chance to be converted to CH_4_ (Figure 3). The proportion of starch degraded in the rumen, the rate of degradation in the rumen, and rumen escaped starch need to be considered when determining the CH_4_ mitigation potential. The complex interactions between the rate of rumen fermentation, pH and the passage rate of starch from the rumen could have resulted in inconsistent results reported in the literature attempting to link the supplementation level of concentrates with the amount of CH_4_ emissions. Rumen passage rate is affected by many factors including supplement concentrates in the diet, whereas it could result in a shift of dissolved hydrogen and consequently rumen fermentation pattern and CH_4_ emissions (20). Another aspect to consider is the effect of feeding concentrates on fibre digestion. Supplementation of large amounts of grain suppresses the degradation of fibre, which will result in less hydrogen produced and, therefore, less CH_4_. For instance, a recent study conducted by Bougouin et al. (119) showed that cows fed with starch-rich diets based on grass silage has comparatively lower enteric CH_4_ emission than fibre-rich diets based on grass silage. The main reasons for reduced methanogenesis may be a reduction of the rumen protozoa population (by 36%) and a shift in rumen fermentation toward propionate at the expense of butyrate. However, increasing the level of starch should be carefully applied as a high amount of starch feeding can lead to ruminal acidosis. According to Nozière et al. (120), the chemical treatment of grains can be used to prevent rumen acidosis by reducing the digestibility of the starch inside the rumen. Furthermore, introducing buffers (e.g., sodium bicarbonate or plant buffering capacity) to starch-based diets reduces the prevalence of rumen acidosis by stabilising rumen pH and improving feed digestion (121, 122).

### Supplementing Feeds Differing in Main Carbohydrates

Hindrichsen et al. (53) compared feeds with different major carbohydrates represented by different substrates including oat hulls (lignified fibre), soybean hulls (non-lignified fibre), apple pulp (pectin), sugar beet pulp (hemicelluloses and pectin), guar gum (galactomannan), Jerusalem artichoke tubers (fructan), molasses (sucrose) and wheat (starch). They were incubated in RUSITEC fermenters mixed with forage (a mixture of maize silage, grass silage and hay) at a ratio of 1:1, on a DM basis, and CH_4_ emissions (mmol CH_4_/g degraded OM) were lower for oat hulls, guar gum and wheat than for molasses, with other supplements intermediate (Table 3). Multiple-regression analysis of CH_4_ emission data with chemical composition revealed a negative correlation with lignin and a positive correlation with total sugars (53). The same diets, except sugar beet pulp and guar gum, were fed to 12 Brown Swiss dairy cows with forage: concentrate ratios of 1: 1 (123). Methane emissions per unit of DMI were only lower for cows fed oat hulls (Table 3), which might have resulted from a lower digestibility due to the high degree of lignification of fibre in oaten hulls. Additionally, CH_4_ emissions per unit of DM were numerically 10% lower for cows fed the Jerusalem artichoke tubers than cows fed wheat. When emissions were expressed as per unit of digested OM, the difference among diets disappeared (123).

**Table 3:** Enteric methane emissions from cows and RUSITEC fed different diets (53, 123).

**Methane**	**Oat hulls**	**Soybean hulls**	**Sugar beet pulp**	**Apple pulp**	**Jerusalem artichoke**	**Guar gum**	**Molasses**	**Wheat**	***P***
Cow									
g/cow/d	330	429	-	351	377	-	382	409	0.109
g/kg DMI	20.7*^*b*^*	25.0*^*a*^*	-	24.8*^*a*^*	23.6*^*ab*^*	-	24.8*^*a*^*	25.3*^*a*^*	0.006
g/kg digested OM	54	56	-	54	49	-	52	55	0.195
RUSITEC									
mmol/g degraded OM	0.92^b^	1.13^ab^	1.24^ab^	1.15^ab^	1.21^ab^	0.99^b^	1.37^a^	1.04^b^	0.004

*DMI, dry matter intake; OM, organic matter*.

*Values in a row with different superscripts differ significantly*.

Methane emissions were 16% higher for sheep fed 1.0 kg molassed sugar-beet pulp (containing 166 g sugars and 166 g cellulose) than for sheep fed 1.0 kg chopped hay (125 g sugars and 302 g cellulose) (124). Methane emissions were 8% higher for lactating dairy cows fed grass-clover silage based diet supplemented with molasses (g/kg diet DM, 250 g starch, 32 g sugar, 300 g NDF) *vs*. wheat grain (g/kg diet DM, 5 g starch, 240 g sugar, 278 g NDF) (125). Giger-Reverdin and Sauvant (126) analysed a CH_4_ emission dataset from male sheep fed 22 different feed classes, which resulted in the separation of feeds into four CH_4_ yield groups being: high—peas and faba beans; medium—grains, sugar beet pulp and soybean meal; low—silages, hays and straw; very low—distillers grains. However, there was substantial variation in CH_4_ yield within feed class, and the proportion of the test feed in the diet and type of basal forage diet were not mentioned.

A recent study conducted by Jonker et al. (127) found that dry cows grazing fodder beet (*Beta vulgaris*) supplemented with perennial ryegrass based pasture silage (6 kg DM/cow/day) produced 28% lower CH_4_ yield (g/kg DM intake) than cows that grazed forage kale (*Brassica oleracea*) supplemented with barley straw (*Hordeum vulgare*) (3 kg DM/cow/day). Fodder beet has a very high concentration of readily fermentable carbohydrates, which may affect rumen fermentation and thereby reduce the CH_4_ emission. Børsting et al. (122) found that feeding sugar beet molasses instead of wheat increased enteric CH_4_ production and yield. This might have been because a high sugar concentration in sugar beet molasses increased the proportion of butyrate in rumen liquid leading to greater H_2_ formation, which will increase the enteric CH_4_ production (20) when the rumen pH is maintained at the normal level (109).

## Conclusions and Recommendations

The different carbohydrates present in different plants may impact the quantity of CH_4_ emissions from ruminants consuming these plants. The detailed compositions and structures of carbohydrates in plants fed to ruminants have not been extensively studied. So far, most carbohydrate information on forage and feeds is based on analysis using the detergent system, which only provides a crude classification of structural and non-structural carbohydrates. Forage brassicas, chicory, and white clover have more readily fermentable carbohydrates (all low starch) and less structural carbohydrates than ryegrass, but the type of carbohydrates in these forages are largely unknown. It is recommended that a comprehensive carbohydrate study is carried out on these forages as these data may help explain variation in CH_4_ emissions observed in animals fed different forages.

Based on evidence from the *in vitro* studies, all carbohydrates, except rhamnose and pectins, may have the same efficiency of being converted to CH_4_. However, rhamnose is generally a minor component in plants. Methyl groups in pectic polysaccharides can be converted to methanol and further converted to CH_4_ in the rumen. Although some *in vitro* studies have suggested that CH_4_ emissions may be lower for some types of carbohydrates, *in vivo* studies have not clearly supported that conclusion. The inconsistent *in vivo* results with soluble sugars make it difficult to speculate if the soluble sugars in forage brassicas contribute to low CH_4_ emissions from sheep fed these forages. Nevertheless, brassicas, chicory, and white clover all contain a large amount of pectic polysaccharides. Compared to ryegrass pastures, brassicas have reduced CH_4_ emissions, while chicory and white clover do not. Since methyl groups in pectic polysaccharides increase CH_4_ emissions, the degree of methyl esterification might make the difference and needs to be elucidated for these forages. How high pectin containing diets and the concentration of methyl group in the diets affect CH_4_ emissions should be studied in the future.

Supplementation of concentrate is considered to reduce CH_4_ emissions due to the associated shift of rumen fermentation. However, this is mainly observed for diets with a high proportion of concentrates (>80%). There are several possible mechanisms for carbohydrates to affect CH_4_ emissions. The fermentation of starch in the rumen produces short-chain fatty acids, which may reduce rumen pH if the rate of absorption and escapement from the rumen is less than the rate of production and subsequently alter the structure of rumen microbial communities. The degradation of starch and structural carbohydrates in the rumen results in different ratios of acetate, butyrate,and propionate. In general, starch promotes the formation of propionate, although sometimes butyrate, which is associated with less CH_4_ production. Structural carbohydrates promote the formation of acetate, which provides hydrogen for CH_4_ production. Diets containing high amounts of fermentable carbohydrates fed at a high feeding level can increase rumen passage rate, which may increase the escape of substrates for microbes to produce hydrogen and lead to lower CH_4_ emissions. The degradation rate of starch and the level of starch intake affect the amount of starch escaping from the rumen. Similar bypass from rumen digestion applies to other polysaccharides and rumen bypass nutrients may occur in ruminants fed fresh forage, especially when the passage rate is high, which could be a result of high levels of feed or water intake. It is recommended that studies are conducted on the sites of digestion of carbohydrates in forages such as brassicas to determine if changes in the sites of digestion are responsible for the mitigation effects measured from this group of forage crops.

## Author Contributions

XS and DP: conceptualisation. XS, AJ, and SM: writing—original draft preparation. XS, LC, AJ, and DP: writing—review and editing. All the authors approved the final version of the manuscript.

## Funding

This work was financially supported by the Pastoral Greenhouse Gas Research Consortium (PGgRc), Wellington, New Zealand, under the Low GHG Feed programme, and by the Department of Science and Technology of Jilin Province, Changchun, China, with a grant (No. 20200602016ZP).

## Conflict of Interest

The authors declare that the research was conducted in the absence of any commercial or financial relationships that could be construed as a potential conflict of interest.

## Publisher's Note

All claims expressed in this article are solely those of the authors and do not necessarily represent those of their affiliated organizations, or those of the publisher, the editors and the reviewers. Any product that may be evaluated in this article, or claim that may be made by its manufacturer, is not guaranteed or endorsed by the publisher.
